# Nutritional Elements in Sleep

**DOI:** 10.7759/cureus.32803

**Published:** 2022-12-21

**Authors:** Harsha Pattnaik, Mikael Mir, Sydney Boike, Rahul Kashyap, Syed Anjum Khan, Salim Surani

**Affiliations:** 1 Medicine, Lady Hardinge Medical College, University of Delhi, New Delhi, IND; 2 Medical School, University of Minnesota Medical School, Minneapolis, USA; 3 Medical School, University of Minnesota Medical School, Minneapolis, MN 55455, Minneapolis, USA; 4 Global Clinical Scholars Research Training, Harvard Medical School, Boston, USA; 5 Research, Global Remote Research Program, St Paul, USA; 6 Critical Care Medicine, Mayo Clinic, Rochester, USA; 7 Research, WellSpan Health, York, USA; 8 Critical Care Medicine, Mayo Clinic Mankato, Minnesota, USA; 9 Anesthesiology, Mayo Clinic, Rochester, USA; 10 Medicine, Texas A&M University, College Station, USA; 11 Medicine, University of North Texas, Dallas, USA; 12 Internal Medicine, Pulmonary Associates, Corpus Christi, USA; 13 Clinical Medicine, University of Houston, Houston, USA

**Keywords:** healthy diet, diet modification, good sleep quality, sleep quality of life, sleep quality & quantity, sleep hygiene, sleep health, nutrition, chrono-nutrition, circadian rhythm

## Abstract

Sleep comprises one-third of our day and plays an integral role in human health and well-being. Many factors influence sleep, with nutrition being a key element that impacts various sleep parameters. Meal-timing through strategies like chrono-nutrition leads to positive sleep outcomes. In addition, consuming a high-protein diet with essential amino acids, low-glycemic-index foods, and certain fruits rich in antioxidants can all contribute to better sleep quality. Other facets of nutrition that can affect sleep outcomes include weight loss and limiting certain nutritional elements such as caffeine, nicotine, and alcohol. In this article, we will shed some light on how some of these factors can play a vital role in sleep quality.

## Introduction and background

Sleep is an integral part of life, a necessary constant that easily accounts for about one-third of our day [1]. Sleep is helpful for resting, healing, consolidating information in memory, and laying the groundwork for the next day’s activities. Sleep is essential for the optimum functioning of the body, including development, repairment, cognition, memory, immune function, psychological state, and overall well-being [2].

Studies have linked decreased sleep duration to declining cognitive function as one ages. However, there is some debate on whether there is such a correlation due to the confounding factor of age being associated with both phenomena [3]. Sleep deprivation is associated with deficits in higher mental function and cognition abilities such as attention, memory, reasoning, and problem-solving across most age domains, but primarily in younger populations as opposed to the elderly [4-6].

Any discussion related to sleep is incomplete without a brief overview of the circadian rhythm and the role that melatonin plays therein. Melatonin is secreted by the pineal gland in a temporal pattern beginning about two hours before bedtime. It is a potent mediator that initiates sleep and is an effective treatment for insomnia [7]. There has been a growing interest in boosting melatonin levels through diet as an alternative to sleeping aids. 

Sleep is influenced by many factors, both intrinsic and extrinsic. Internally, genetics, hormones, and disease states of the mind and body influence sleep. Environment and nutrition play an essential role in sleep. Discussion around sleep hygiene highlights this role, as proper temperature [8], darkness [9,10], silence [11], and comfort, along with sleep-conducive behaviors before bedtime, are factors that influence the quality as well as the quantity of sleep [12].

Various disease states can lead to sleep problems, and sleep disorders themselves can lead to cardiovascular [13] and neuropsychiatric issues [14]. Studies suggest that these relationships are bidirectional and require more research to elucidate how they are linked. Sleep disorders are a global challenge [15]. The excessive use of technology and media has led to poor sleep hygiene [16,17]. The prevalence of insomnia and other sleep disorders has been on the rise and is a cause for concern [18,19]. Similarly, there has been a rise in various mental health issues, which are possibly associated with excessive use of technology [14,20-22]. Studies have also shown correlations between poor sleep and hypertension [23-25], obesity [25-27], diabetes [28,29], dyslipidemia [30,31], and metabolic syndrome [32]. 

The modern human diet, which is calorie-dense with added preservatives but lacking essential micro and macronutrients, is also responsible for the rise in sleep disorders in recent years. We plan to elaborate on this issue in this article.

## Review

Chrono-nutrition

Chrono-nutrition [33,34] is a multidisciplinary approach to nutrition that studies **the impact of food intake on health and the timing of eating, affecting our body clock and sleep-wake correlation**. It generally addresses three considerations: irregularity of meal timings, frequency of meals, and body-clock time (Table 1). These components could affect the circadian rhythm, influencing the sleep-wake cycle and a person’s metabolic health. Our circadian rhythm determines the most efficient timing for food digestion and metabolism. Consuming food outside of these time frames often leads to poor digestion, which can interfere with sleep. Closer proximity of dinner time to sleep time, especially if less than two hours, leads to poor sleep quality [35-39].

**Table 1:** Factors of chrono-nutrition and how they affect sleep quality.

Chrono-nutrition Consideration	Contributing Factor	Effects on Sleep
Meal timing	Food intake when circadian rhythms in metabolic processes are not optimal for nutrition	Poor digestion leading to worse sleep quality [33]
	Consuming a meal less than 2 hours before sleep time	Poor sleep quality [35-39]
	Consuming a major portion of calories at night	Alterations in the gut microbiota to a pro-inflammatory state leading to misalignment of the circadian rhythm and poor sleep [38,40,41]
	Time-restricted eating	Improvement of sleep quality (independent of weight loss) [42]
Meal frequency	Consistent meals 3x/day	Better sleep quality [43]
Body-clock time	Evening chronotype	Worse sleep quality [44]

Studies have shown that meal timing often influences gut microbiota. Research on shift workers, who often consume a major portion of their calories at night, has shown significant changes in the composition of their gut microbiome’s composition, which is linked to the misalignment of circadian rhythm leading to poor sleep [38,40,41]. More research is needed to explore this potentially critical link further. Studies on chrono-nutrition have demonstrated that people who have a more irregular meal routine have a higher risk of obesity and metabolic syndrome when compared to those with a more regular meal routine [38,45,46].

Time-restricted eating entails consuming the major portion of one’s daily calorie intake within a limited window of time, which is considered an effective weight-loss strategy. However, aside from the indirect effect it has on sleep through mitigating obesity, it is also shown to positively affect sleep outcomes [47,42] independent of it causing weight loss and improving cardiometabolic health [35,43].

Obesity and sleep

A bidirectional relationship exists between obesity and sleep. Short sleepers often make poor nutritional choices and have higher caloric intakes compared to people who sleep >7 hours a night. Short sleepers, defined as sleeping <7 hours/night, consume a less diverse selection of foods with lower protein and fiber intake than those sleeping longer [48]. Intervention studies have reported higher snacking behavior [43,49] during periods of sleep restriction when compared to normal sleep [50]. There is also an increased craving for high-glycemic-index foods with high carbohydrate content, particularly in the afternoon and evening hours. Additionally, fat is reported to be the macronutrient of choice during periods of sleep restriction relative to periods of normal sleep duration [51,52]. These studies show how sleep and, most importantly, sleep restriction affect the food choices one makes. Poor sleep has also been shown to affect leptin and ghrelin levels. Short sleep is associated with increased ghrelin levels and decreased leptin levels, leading to more hunger and less satiety, leading to increased calorie intake [53]. This constant lack of sleep leading to poor food choices, in the long run, can lead to obesity and its associated complications [13].

On the other hand, obesity itself can lead to poor sleep quality. Obstructive sleep apnea (OSA) is quite common in obese individuals [25,52,54,55]. OSA is associated with poor sleep quality, fragmented sleep, nighttime awakenings, and increased daytime somnolence, as well as an increased risk for cardiometabolic diseases, mental illness, and impaired cognitive skills [56].

Poor nutritional and lifestyle choices can lead to obesity, which can lead to OSA and, consequently, poor sleep quality. Consistent consumption of saturated fats and high-glycemic-index carbohydrates, in addition to a lack of exercise, can lead to this problem. Interestingly, independent of OSA, obesity itself is associated with poor sleep quality, increased need for sleep, and increased daytime sleepiness [27]. Healthier lifestyle modifications can help ensure good, restful sleep. Weight loss can improve sleep outcomes in obese people, which needs to be further explored in larger-cohort studies.

Sleep-promoting nutritional elements

Melatonin-Boosting Foods

Melatonin is a hormone produced in the pineal gland and plays an important role in sleep onset and circadian rhythm [7]. It has been identified in plants [57,58], meats [59], mushrooms [60], and some fruits such as pineapples, oranges, and bananas [61]. Given melatonin’s potent role in sleep, it is considered a quintessential sleeping aid that is available over the counter. It significantly improves sleep quality and decreases sleep onset latency in insomnia without the morning-after grogginess associated with other common classes of sleep medication [62]. Melatonin is naturally found in various plants and animal food sources in nature. It is theorized that the consumption of melatonin-rich foods can increase the serum melatonin concentration [61] and antioxidant capacity in human beings, which would assist in good sleep; these foods are now considered promising nutraceuticals [63]. Certain varieties of grapes [64] and cherries [65] are being used to create phytochemical-based nutraceuticals to promote good sleep, which can be reinforced by consuming a portion of raw fruits and vegetables and lean meats in one’s everyday diet. 

A pilot study demonstrated that, when consumed twice daily, fresh tart cherry juice effectively reduced sleep latency and improved insomnia in those >50 years of age. Studies have reported increased urinary melatonin concentrations upon consuming tart cherries, buttressing that they have a high dietary melatonin concentration and phytonutrient profile [66-69]. Tart cherries also exhibit anti-inflammatory characteristics that may improve sleep quality. One study showed that tart cherries might mediate improvements in sleep quality by minimizing oxidative damage due to the abundance of antioxidants found in cherries [66,70]. 

Serotonin and Tryptophan

Kiwifruit is considered a good source of serotonin, which is known to help to promote better sleep and plays a modulatory role in the sleep-wake cycle [71]. One study showed that consuming two kiwis an hour before bedtime improved serotonin levels, thus helping to increase total sleep time and sleep quality, with fewer post-sleep-onset awakenings in those experiencing sleep disturbances [72]. Kiwi is rich in vitamins C and E and has a high antioxidant capacity, which is protective against free-radical damage; it is also a source of folate, which, when deficient, is related to insomnia [73]. One animal study showed decreased melatonin secretion in the folate-deficient group [74]. Folate is destroyed by cooking, but folate-containing fruits like kiwi are often consumed in their raw form, which preserves their nutritive value.

Tryptophan is necessary for serotonin synthesis, and tryptophan depletion leads to sleep disturbances through the serotonergic pathway [75]. It has been reported that significant decreases in total sleep time, longer sleep onset latency, and sleep efficiency occur after tryptophan restriction [76]. Tryptophan deficiency is also associated with increased wake periods [77]. Hence, the consumption of tryptophan-rich food items like cereals and a high-protein diet containing tryptophan can assist in improving sleep outcomes.

Fatty Fish and Seafood 

A high intake of fish and vegetables has a positive effect on sleep [78,79]. Fatty fish is rich in vitamin D and omega-3 fatty acids. Vitamin D deficiency is associated with sleep disorders [80], and omega-3 fatty acids are associated with positive sleep outcomes [81]. Hence, having a diet rich in fatty fish can play a role in improving sleep quality. However, one study in preschool children reported no significant improvement in sleep with a diet consisting of fatty fish, but this finding is limited by the study methodology, which relied on parents’ subjective opinions instead of objective methodology such as polysomnography or laboratory tests [82]. Thus, further studies are needed to explore the relationship between fatty fish and sleep. Seafood such as oysters and fish are rich in zinc. One study examined the consumption of zinc-rich oysters and astaxanthin-containing krill and found improvements in global sleep quality scores in terms of the Pittsburgh Sleep Quality Index and sleep onset latency [83].

Milk

Milk is widely considered a sleep-promoting food in many cultures worldwide. Children are often given milk with turmeric before bedtime in certain Eastern cultures. Cow milk has high levels of tryptophan and is conducive to sleep [84], whereas curcumin found in turmeric has been shown to increase non-rapid eye movement sleep via histamine H1 receptor blockade in rats [85]. Additionally, turmeric has been shown to have a protective effect on neuronal loss due to chronic sleep deprivation through antioxidant properties in animal studies [86], but human studies are needed to establish such claims. The general consensus is that the intake of enriched milk and dairy products leads to anti-inflammatory and antioxidant changes in the brain-gut-microbiome axis and thus promotes good sleep [87]. Milk is a natural source of melatonin, and milk obtained through milking at night contains a higher content of melatonin and has a sleep-promoting effect [88]. A systematic review showed that milk and dairy products, when included in a well-balanced diet, are effective in improving sleep quality.

However, it is difficult to consider milk as the sole reason for good sleep independent of other dietary factors. Various studies have been conducted to assess the role of these products in influencing sleep duration and quality. However, whether milk and dairy consumption has a positive effect on sleep remains unclear due to the heterogeneity of research methodologies and techniques, although several beneficial effects on different sleep parameters were seen (i.e., electroencephalography, actigraphy, and subjective assessment) [87]. In one study, when *Lactobacillus helveticus*-fermented milk was used in elderly people, there was an improvement in sleep efficacy and a reduction in the number of episodes of awakening, which were measured by means of actigraphy and a sleep questionnaire [89]. A combination of exercise and milk consumption is linked to improving sleep onset latency in older adults [90].

High- and Low-Carbohydrate Diets

Carbohydrates are the main food group in staple diets across the world. They serve as the main source of fuel for our body to function and, hence, influence our sleep. As per a meta-analysis studying the effect of low versus high dietary carbohydrate content on sleep parameters, the former was associated with shorter time before falling asleep and higher sleep efficiency, which is defined as the percentage of time spent asleep relative to the total time in bed [91]. Not only is the amount of carbohydrates important in influencing sleep architecture but also the type of carbohydrates, especially the glycemic index, as well as the timing of their consumption. One study showed that sleep onset latency was significantly reduced after the consumption of high-glycemic-index meals four hours before bedtime compared to the consumption of low- or high-glycemic-index meals closer to bedtime [92]. Another study on middle-aged Japanese women found that a high intake of confectioneries and noodles (i.e., high glycemic index and simple carbohydrates) and a lack of vegetables in the diet was associated with poor sleep quality [78]. Carbohydrate quality is important in determining sleep quality. Studies report low carbohydrate intakes in those with insomnia symptoms [93,94] but relatively higher intakes of sweets [78] and fats [93,94].

Specific diets

Mediterranean Diet

The Mediterranean diet is rich in legumes, fruits, whole grains, fish, and vegetables. Consumption of a Mediterranean diet with a decrease in red meat and alcohol intake has been associated with improved sleep outcomes in various populations, from teenagers to older adults [95-97]. It has been associated with improvements in parameters such as difficulty initiating sleep, difficulty maintaining sleep, and early morning awakening in older adults [98]. This diet avoids high fat intake, which has been associated with sleep disorders [93]; hence, consuming this diet has been shown to improve sleep quality effectively.

DASH Diet

The Dietary Approach to Stop Hypertension diet is similar to the Mediterranean diet, focusing on higher intakes of vegetables, fruits, fish, and nuts and lower intakes of red and processed meat, refined carbohydrates, sugar, and salt. The DASH diet has been shown to have a negative correlation with insomnia [99] and could serve as an effective lifestyle modification to overcome sleep disorders.

Nutritional elements impairing sleep

Caffeine 

Caffeine is one of the most widely consumed stimulants in modern times. It is found in common beverages like coffee, tea, carbonated drinks, and energy drinks, as well as chocolates. Close to 90% of US adults consume caffeinated items, with the average daily intake being 211 mg - equivalent to about two cups of brewed coffee [100,101]. Caffeine is an adenosine-receptor antagonist; hence, it increases arousal, wakefulness, and alertness and counteracts fatigue and sleep [102]. Some people experience effects like anxiety and panic attacks [103] due to these stimulatory effects when caffeine is consumed in higher doses, which may interfere with sleep. Daytime consumption of caffeinated products causes a decrease in the main metabolite of melatonin, 6-sulfatoxymelatonin, at night [104], which disrupts the circadian rhythm and negatively affects sleep onset and quality. Consistently high caffeine use chronically impairs sleep patterns [105,106]. When consumed in the morning, caffeine levels in the body fall to less than one-fifth of the initial spike 16 hours later. However, one study found that morning caffeine use affected sleep efficiency and total sleep time negatively [107]. On the contrary, another study of an Ecuadorian village found no significant effect of daytime caffeine use on nighttime sleep [108]. Another study found that 400 mg of caffeine - equivalent to four cups of coffee (a high dose considering the average caffeine consumption) - taken 0, 3, or even 6 hours before bedtime significantly disrupted sleep. Caffeine was shown to reduce total sleep time by more than one hour, even when consumed six hours before bedtime [109]. Another study showed that caffeine consumption 30 minutes before bedtime produced effects similar to insomnia, and there was a dose-dependent relationship between caffeine intake and sleep disruption [110].

Nicotine

Nicotine is a stimulant that is found in tobacco products. Cigarettes, e-cigarettes, and vapes are commonly used in modern times [111]; hence, it is important to consider the effect of nicotine on sleep. Nicotine consumption is known to increase sleep latency and sleep fragmentation and decrease sleep efficiency and quality [112]. Smokers have a higher risk of developing sleep-related issues, and adverse impacts on sleep can be somewhat mitigated by removing nicotine consumption [113]. Nicotine use within four hours of bedtime leads to increased sleep fragmentation and awakenings [114].

Alcohol

Alcohol is a central nervous system depressant. Acute consumption of large amounts of alcohol prior to sleep leads to decreased sleep onset latency, but later on, this behavior disrupts the normal sleep architecture, leading to poor sleep quality and increased sleep fragmentation and nighttime awakenings, culminating in reduced total sleep time [115]. Chronic alcohol use is also associated with chronic sleep disturbance, and there is an association between alcohol use disorder and insomnia [116]. Alcohol consumption within four hours of bedtime leads to increased sleep fragmentation and awakenings; therefore, its use should also be curtailed at least four hours before bedtime [114].

Reflux-Inducing Food

The consumption of fried fatty foods, spicy foods, and junk foods close to bedtime can lead to acid reflux and heartburn, which can cause nighttime awakenings and poor sleep quality [117]. One must opt for vegetables and whole foods and alternatives to deep-frying like roasting, air-frying, or boiling to prepare food for dinner to avoid gastric reflux while sleeping. It is also recommended to eat dinner at least two hours before bedtime [35-39] and to not immediately lie down after consuming a meal.

## Conclusions

Studies show that diet influences our sleeping habits more extensively than previously thought. Nutrition plays an important role in health and has a bidirectional relationship with sleep. Exercise combined with good nutrition leads to a restoring and rejuvenating night’s sleep. Timing meals through the strategies of chrono-nutrition, consuming a high-protein diet rich in essential amino acids like tryptophan, consuming low-glycemic-index carbohydrates, and consuming fruits that are rich in sleep-promoting properties and antioxidants like cherries and kiwis are helpful approaches to improving sleep. Losing weight can also help improve sleep outcomes, and positive dietary changes are essential. Removing nicotine, alcohol, and caffeine is important for sleep, but in most cases, limiting consumption to at least four hours before bedtime will lead to better sleep.
